# Fragmentation of Cu_2_O Oxides Caused by Various States of Stress Resulting from Extreme Plastic Deformation

**DOI:** 10.3390/ma18081736

**Published:** 2025-04-10

**Authors:** Małgorzata Zasadzińska

**Affiliations:** Faculty of Non-Ferrous Metals, AGH University of Krakow, al. Mickiewicza 30, 30-059 Krakow, Poland; malgozas@agh.edu.pl

**Keywords:** extreme plastic deformation, drawing and rolling process, cold metal working, Cu-ETP, Cu_2_O

## Abstract

The development of microelectronics results in higher demand for copper microwires and thin foils. Higher demand requires conducting research to obtain knowledge on the influence of extreme plastic deformation on materials’ susceptibility to plastic processing without the loss of coherence. One of the key factors contributing to rupture during the plastic deformation of copper is the presence of micrometer-sized, eutectic Cu_2_O oxides, which are weakly bound to the copper matrix. These oxides are formed during the metallurgical stage of wire rod copper manufacturing. Copper wire rod of the ETP (electrolytic tough pitch) grade was subjected to wire drawing followed by cold-rolling. Applying different states of stress during plastic deformation (wire drawing, cold-rolling, and upsetting) made it possible to specify the conditions required for Cu_2_O oxides’ fragmentation due to the extreme total deformation. Qualitative and quantitative analyses of the Cu_2_O oxides’ evolution and fragmentation as the plastic deformation progressed were the main focus of this paper. It was determined that major fragmentation occurred during the initial stages of plastic deformation. Applying further extreme deformation or changing the state of stress during plastic deformation did not facilitate the continuation of fragmentation. It was only their shape that was becoming elongated.

## 1. Introduction

There are numerous widely used copper grades; however, the most common and used is ETP-grade copper (electrolytic tough pitch copper), which is produced in metallurgical processes of high-purity cathodes obtained by electrolysis. ETP-grade copper has standardized contents of oxygen with a maximum value of 400–600 ppm, depending on the origin of the standard. The excellent deformability of copper combined with its high electrical conductivity and tensile strength [1,2,3] result in immense demand and advanced processing technology worldwide. The most common application of ETP-grade copper is as the raw material input for the manufacturing process of wires and microwires intended for cables and conductors [4], as well as several others, such as trolley contact wires, busbars, etc. [5,6].

**Figure 1:**
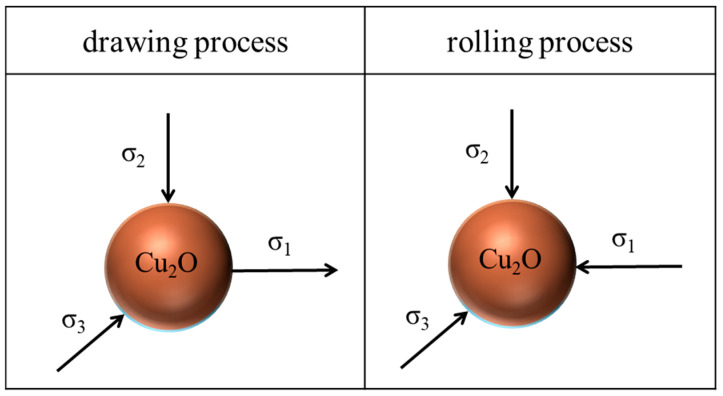
State of stress affecting Cu_2_O oxide during the drawing process (**left**) and the rolling process (**right**)—own work.

Researchers indicate that oxygen has a significant influence on the copper matrix. They report, among others, that oxygen affects the electrical conductivity, kinetics of recrystallization, and risk of hydrogen disease [5,7,8,9] or may even limit the deformability of copper due to the presence of eutectic Cu_2_O oxides in the copper matrix [10,11]. Cu_2_O oxide particles are present in the matrix throughout the whole technological process, beginning with the melting of cathodes, continuous casting and crystallization of the strand, hot-rolling to the form of the wire rod, and finally the cold-drawing process to obtain wires and microwires [12]. Mysik et al. [13] stated that Cu-Cu_2_O eutectic may function as the stress concentration points at the grain boundaries and, as a result, may lead to fractures at further stages of copper wire processing. Similar conclusions were presented by Zhao et al. [14], with the authors claiming that the presence of second-phase particles of Cu_2_O oxides resulted in the formation of microstructural defects caused by the dispersion hardening mechanism. Due to being a ceramic material, Cu_2_O oxide is significantly harder than copper, and its precipitates are incoherent to the matrix [15,16]. Research papers indicate that the presence of voids and/or inclusions is the most common reason of limited deformability in the wire drawing process [17,18,19,20]. Indeed, the authors in [21,22,23,24,25] claim that the vast majority of technological ruptures and breaks during the wire drawing process of copper happen as a result of inclusions, including the presence of Cu_2_O oxides. Therefore, it may be assumed that Cu_2_O oxides initiate the formation of wire ruptures and, as such, are one of the main threats to the cold-drawing process and cause of limited deformability of ETP-grade copper. Research results presented by Zasadzińska and Knych [26] proved that Cu_2_O oxides fragmentated only during the initial stages of the diameter reduction in ETP-grade copper wire rod. The oxides were reduced from the original size of up to 5 µm to below 1 µm. However, further fragmentation did not occur regardless of the applied deformation. When considering the cold-rolling process, analyses of Cu_2_O oxides’ evolution were conducted only regarding thick plates and sheets [14,27], whereas no research was performed on thin strips and foils. The way Cu_2_O oxide particles behave might be entirely different due to the fact that drawing and rolling processes have significantly different states of stress [28,29]. Both are triaxial, but in terms of the drawing process, the state of stress is biaxial compression with dominating uniaxial tension, while in the rolling process it is triaxial compression. Figure 1 presents a schematic representation of the stress components affecting Cu_2_O oxide in the plastic zone of the drawing die and the rolling process.

Therefore, knowledge of the presence, behavior, and evolution of the shape and size of the Cu_2_O oxides’ inclusions during the processing of ETP-grade copper is crucial when considering products of small cross-sections such as microwires and thin foils. Their undisturbed manufacturing process might be endangered when extreme deformation is applied. Lack of this information might be the microscopic or even molecular cause for uncontrollable macroscopic process failures. The issue is especially important due to the miniaturization of electronics and the introduction of copper nanowires to nanoelectronics, where the presence of Cu_2_O oxides might be a significant obstacle [30,31,32]. Hence, the aim of this paper is to determine the influence of the various states of stress on the evolution and morphology of Cu_2_O oxides by subjecting ETP-grade copper to extreme deformation in cold-drawing and cold-rolling processes, as it is a significant, existing knowledge gap.

## 2. Materials and Methods

Reaching the assumed aim of this paper was possible with the use of a laboratory bench drawing machine (RaQun, Gorlice, Poland) and a laboratory four-high rolling mill (Radius, Katowice, Poland). Firstly, an ETP-grade copper wire rod with a diameter of 8 mm was subjected to 40 draws to obtain a 0.2 mm diameter wire. Drawing dies with an optimal copper drawing die angle of 9° and a constant unit coefficient of deformation λ = 1.2 were used [33]. At this point, the true strain of the final 0.2 mm diameter wire was εtr = 7.33. The obtained wires were subjected to 3 rolling passes. Thus, a thin foil with a thickness of 0.05 mm was obtained, and its true strain was εtr = 10.15, with a total deformation of εt = 99.99%. Materials subjected to these extreme deformations were analyzed using a Hitachi SU-70 scanning electron microscope (SEM) (Hitachi Ltd., Tokyo, Japan). The SEM used in this study was equipped with an EDS analyzer, which was used during the identification of Cu_2_O oxide inclusions and oxygen content in the ETP-grade copper. SEM analysis was conducted using backscatter electrons (BSEs) with the accelerating voltage set to 15 kV, the working distance set to 10 mm, and the maximum magnification set to 10,000×.

Additional research was conducted using a 3 MN hydraulic press (Hydromet, Bytom, Poland) in order to determine the value of the compressive force necessary for further fragmentation of Cu_2_O oxide particles. Upsetting tests were conducted using ETP-grade copper samples with a volume of 1200 mm^3^ machined from a wire rod. Samples were subjected to compressive forces of 250, 500, 1000, and 2000 kN in parallel and perpendicular directions to the wire rod axis. The analysis of the obtained samples was conducted using the same SEM with the same parameters as described above.

## 3. Results

**Figure 2:**
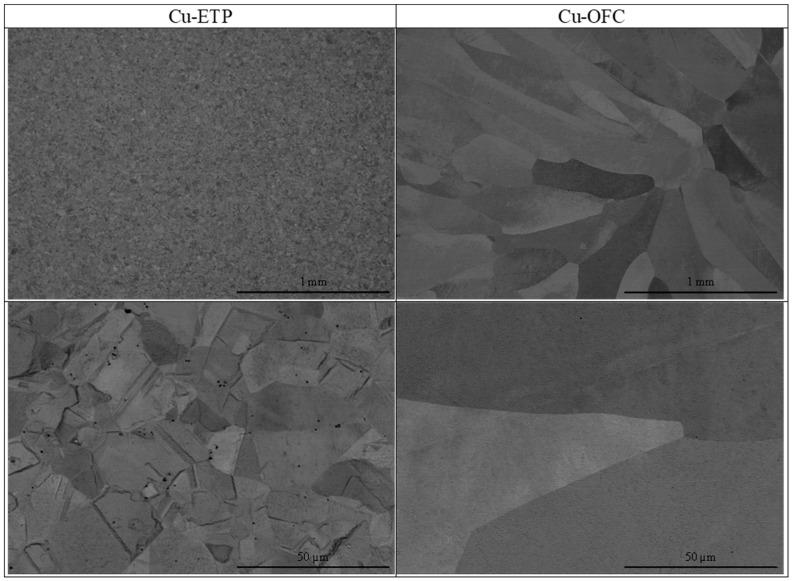
Microstructure images of ETP-grade copper wire rod and OFC rod.

This study on oxygen’s influence on the microstructure of copper began with the analysis of the wire drawing feedstock, i.e., wire rod of the ETP-grade copper and the oxygen-free copper rod (OFC) of the same diameter (8 mm) as a reference. The manufacturers declared in their certificates of quality that ETP-grade copper had 160 ppm of oxygen, while the level of oxygen in the OFC was a maximum of 2 ppm. The microstructure analysis conducted at the cross-section of both materials is presented in Figure 2. The distinction between the materials is clear and shows the significant difference between these two copper grades and their manufacturing processes [34]. The ETP-grade copper has a fine and homogeneous structure typical of dynamic recrystallization. The grain size is certainly lower than 50 µm. Cu_2_O oxides are present in the interdendritic spaces and grain boundaries. On the other hand, OFC shows a grain structure typical of casting materials with oriented and much larger grains in comparison to ETP-grade copper. No Cu_2_O oxides were observed, and therefore, no fragmentation could occur. Therefore, further analyses were conducted solely on ETP-grade copper.

**Figure 3:**
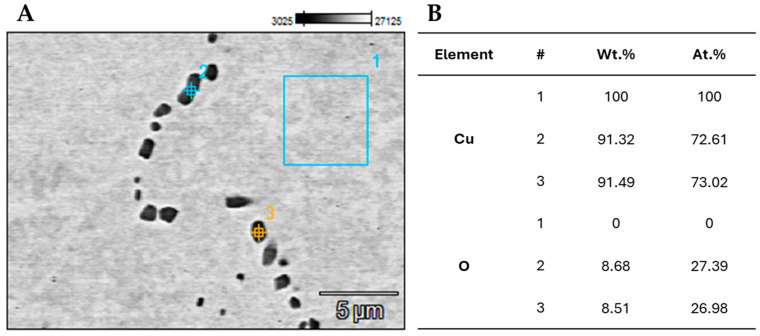
EDS analysis of ETP-grade copper material: (**A**) microstructure with selected points and area; and (**B**) chemical composition in the corresponding points and area.

In order to accurately recognize and confirm the presence of Cu_2_O oxide inclusions, an analysis of the microstructure with the use of EDS (energy-dispersive X-ray spectroscopy) was conducted. The analysis was performed on the ETP-grade copper at the points and area presented in Figure 3A. The chemical composition at the marked spots and area is presented both in weight % (Wt. %) and atomic % (At. %) in Figure 3B. Selected points 2 and 3 show a clear presence of oxygen, suggesting that the black spots are Cu_2_O oxides. There is no oxygen in the selected area 1, where the chemical composition is 100% copper.

**Figure 4:**
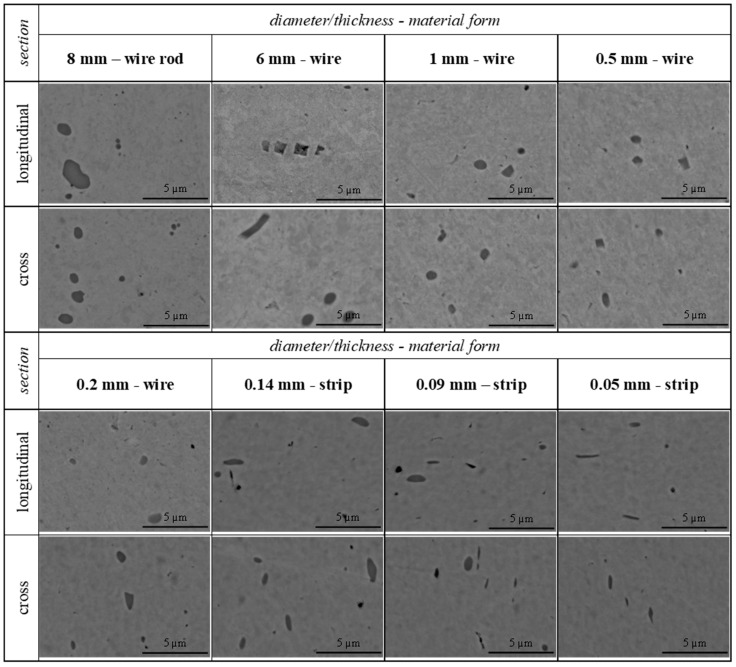
Evolution of the shape and dimensions of Cu_2_O oxides due to the deformation applied in the wire drawing and cold-rolling processes of an ETP-grade copper wire rod.

The next stage of the research included wire drawing and further cold-rolling of the copper wire rod at the ETP grade. Due to the extreme plastic deformation applied to wires and strips, it was determined using SEM (scanning electron microscope) images that, as the deformation progressed, the geometry of the Cu_2_O oxides changed. Cu_2_O oxide crystals were oval and reached a size between 3 and 5 μm when considering the original wire rod. The observed fragmentation and changes in the shape of Cu_2_O oxides were the most intense during the initial stages of deformation (up to the fifth drawing die), when the wire had a diameter of 5–7 mm. As presented in Figure 4, fragmented parts of the Cu_2_O oxides measuring around 1–2 μm were characterized by sharp edges. The copper matrix fully surrounded the Cu_2_O oxides, and the spaces formed between the parts were tightly filled. The distances between the individual parts of the oxides were small, and their original shape could be easily identified. Applying further deformation did not favor fragmentation. The spaces between individual oxides expanded due to the increase in the amount of deformation. The copper matrix was evenly filled with the Cu_2_O oxides, as no segregation towards the axis or surface of the wires was observed.

The wire drawing process was performed down to the diameter of 0.2 mm, and these wires were further subjected to cold-rolling. The extreme deformation and different states of stress prevailing inside the roll gap influenced the shape and size of the Cu_2_O oxide particles. Existing inclusions were characterized by an elongated and distorted shape in comparison to the particles observable after only the wire drawing process. However, no further fragmentation of Cu_2_O oxides was observed. Exemplary images of the evolution and morphology of Cu_2_O oxides throughout the wire drawing and rolling processes of ETP-grade copper are presented in Figure 4.

**Figure 5:**
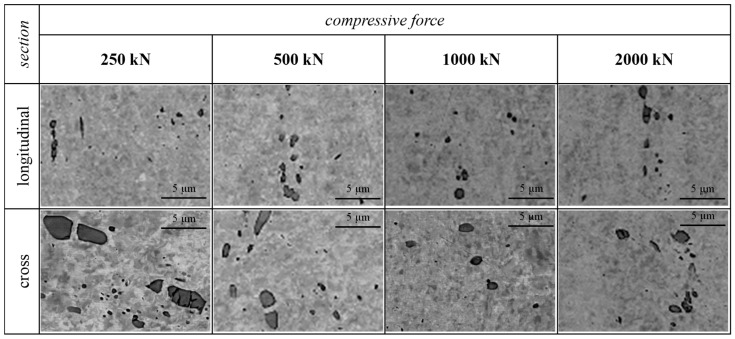
Evolution of the shape and dimensions of Cu_2_O oxides due to the deformation applied in the upsetting test of an ETP-grade copper wire rod.

Since no fragmentation of Cu_2_O oxides occurred during the cold-rolling process of ETP-grade copper wires, this might suggest that the necessary force for that to happen was not achieved. A hydraulic press was used in the upsetting test of the initial wire rod to determine the value of force required to further fragmentate the inclusions. The values of 250–2000 kN of compressive force were applied both at the longitudinal (parallel to the axis) and cross-section (perpendicular to the axis) directions. The results presented in Figure 5 show that a 250 kN compressive force was enough for the Cu_2_O oxides to fragmentate. Further increases in force values resulted in a slight reduction in the particle size, but no subsequent fragmentation occurred.

The qualitative and quantitative analyses conducted in this study showed that compressive forces led to the severe fragmentation of Cu_2_O oxides. As opposed to the drawing process, in the upsetting test, fragmentation of a similar degree was obtained over a single operation, without using several drawing dies. After recalculating, the force applied to stress the material would have to have between 3.2 GPa and 25 GPa of stress. Unfortunately, even though the state of stress during the discussed rolling process was also compressive, the values of stress mentioned above would not have been possible to reach in the cold-rolling process of ETP-grade copper.

## 4. Discussion

Previously published papers [35,36,37] indicate that severe plastic deformation of copper leads to significant strain hardening and may consequently cause the loss of cohesion during plastic deformation of the material. An additional factor contributing to the loss of coherence during metal working processes is represented by the inclusions in the copper matrix, such as Cu_2_O oxides [38].

It is known that oxygen dissolves poorly in copper. It forms two main types of oxides, i.e., copper (II) oxide CuO and copper (I) oxide (Cu_2_O). At elevated temperature up to 380 °C, the CuO compound is formed, while, above 380 °C, it is Cu_2_O oxide [39,40]. ETP-grade copper wire rods are obtained in the continuous casting and rolling process. Both metallurgical synthesis and hot-rolling are performed in temperature ranges exceeding 380 °C, as the final wire rod has a temperature of around 500 °C before cooling to room temperature [41]. Considering the Cu-O phase diagram [42], it may be stated that, as the temperature during solidification decreases below the eutectics’ temperature, the structure will show not only copper crystallites but also a eutectic system. The rapid cooling of copper with the oxygen content below 0.008 wt. % would form a single-phase supersaturated solution of copper with oxygen. However, when slow cooling is applied or the oxygen content is higher, the Cu_2_O oxides would precipitate from the solution [43].

SEM analysis of the materials obtained in the wire drawing process proved that the fragmentation of Cu_2_O oxides occurred only during the initial stages of the process. These initial stages included the first five drawing dies and the diameter reduction from the initial 8 mm down to 5 mm. The spherical shape of the oxide particles with dimensions up to 5 µm was changed into an indeterminate shape with dimensions up to 2 µm. The fragmented Cu_2_O oxides often had sharp edges and almost rectangular shapes, which might indicate brittle cracking. The phenomenon of oxide fragmentation is positive and desired from the point of view of the wire drawing process. The fragmentation of inclusions lowers the risk of ruptures associated with the volume fraction of Cu_2_O oxides in the cross-section of the microwires and, prospectively, nanowires, especially since inclusions facilitate ruptures by weakening the coherence of the copper matrix [44]. Since the diameter of the nanowires is much below 1 µm, it is essential that further fragmentation of Cu_2_O oxides occurs [30,31,32]. Such reasoning was confirmed by Blicharski [45], who stated that inclusion particles should be fine, homogeneously distributed inside of the matrix, and without sharp edges, as these facilitate the nucleation of cracks. The issue of the limited deformability of copper wires as a result of the presence of inclusions was previously addressed by Norasethasopon and Yoshida in [46]. Their analysis included finite-element method simulations of the multi-stage wire drawing process with regard to the influence of the diameter and length of the inclusion (tungsten carbide). They concluded that the diameter of the inclusion had the largest impact on the recorded stress values. According to their results, when the ratio of the diameter of the inclusion to the diameter of the wire is higher than 0.75, in almost all cases, it leads to the formation of chevron cracks and/or wire ruptures. They emphasized that the length and shape of the inclusions were important but had a secondary impact on the process.

It is stated in scientific papers [47,48] and generally available empirical knowledge that ceramic materials are characterized by a higher resistance to compressive forces than to tensile forces. The state of stress inside of the approach angle of the drawing die during the axi-symmetric wire drawing process is characterized by biaxial compression (radial and circumferential) with dominating uniaxial tension parallel to the axis of the wire [28,29]. As the deformation progresses, the metal matrix is being strengthened, and the pressure of the metal on the walls of the drawing die increases. The combination of these factors with the tensile stress causes the fragmentation of Cu_2_O oxides during the initial stages of the wire rod drawing process. However, these mechanisms do not function so well below the diameter of 5 mm. Therefore, the cold-rolling process was introduced in the final stages of manufacturing microwires and thin foils in order to maximize deformation and thus create conditions for the further fragmentation of Cu_2_O oxides. Creating such conditions would be beneficial for the development of manufacturing processes of thin copper products, which would facilitate the progress of nanoelectronics. The cold-rolling process is also characterized by a triaxial state of stress. However, it differs from the wire drawing process as it is triaxial compression with no tension. This should provide conditions for the further fragmentation of Cu_2_O oxides instead of elongating them. Unfortunately, the extreme deformation provided during the cold-rolling process did not activate further fragmentation. Possibly, the forces during the process were not high enough to facilitate fragmentation. The probability of achieving the necessary forces to fragmentate Cu_2_O oxides in cold-rolled wires and thin foils would be higher with further thickness reduction (below 0.05 mm). However, this was impossible with the laboratory rolling stand.

## 5. Conclusions

Based on the obtained results and their analysis regarding the fragmentation of Cu_2_O oxides caused by various states of stress resulting from extreme plastic deformation, the following conclusions were made:

Fragmentation of Cu_2_O oxides occurs during the drawing process of ETP-grade copper wire rods. However, it is most significant during the first stages of deformation, i.e., when the first five drawing dies are applied. It was noted that, below the diameter of 5 mm, further fragmentation did not occur, and the size of the individual Cu_2_O oxides was approximately 1 μm.

During the wire drawing process of microwires, the tensile stress inside of the approach angle of the drawing die is not high enough to overcome the tensile strength of the Cu_2_O oxides. Therefore, particles become elongated and do not fragmentate. The lack of fragmentation is unfavorable due to the increasing ratio of the size of the inclusion to the diameter of the micro- and, prospectively, nanowires. When the ratio is too high, it leads to wire rupture.

Applying plastic deformation with different states of stress in the cold-rolling process did not facilitate the further fragmentation of Cu_2_O oxides. Similarly to the wire drawing process, it was impossible to reach the necessary conditions for the further fragmentation of the oxide particles even when extreme total deformation was applied.

## Data Availability

The original contributions presented in this study are included in the article material; further inquiries can be directed to the corresponding author.
